# Antistreptococcal treatment of psoriasis: a systematic review

**DOI:** 10.1007/s00403-024-03051-8

**Published:** 2024-06-08

**Authors:** Beau Sitton, Trent Walker, Rohan Mital, Vamsi Varra, Jessica Kaffenberger

**Affiliations:** Department of Dermatology, The Ohio State University Wexner Medical Center, 540 Officenter Center Place, Suite 240, Columbus, OH 43230 USA

**Keywords:** Psoriasis, Streptococcus, Guttate psoriasis, Tonsillectomy

## Abstract

Streptococcal infections may contribute to psoriasis development, and antistreptococcal treatments are considered potential therapies, but their effectiveness remains uncertain due to limited systematic evidence. Our objective was to analyze antistreptococcal therapies' effectiveness in improving psoriasis. We conducted a systematic review following PRISMA guidelines, evaluating antistreptococcal treatment efficacy in psoriasis patients from PubMed, Scopus, and Embase databases until August 14, 2022. Eligible studies included psoriasis patients undergoing antistreptococcal therapy, regardless of demographics or psoriasis type. 50 studies (1778 patients) were analyzed, with penicillins/aminopenicillins as the most studied antibiotics (21 studies), showing mixed outcomes, some reporting significant improvement in guttate psoriasis, while others showed no significant difference. Rifampin demonstrated positive results in most of ten studies, and macrolides showed varying effectiveness in two studies. Tonsillectomy in 14 studies (409 patients) mainly focusing on guttate and chronic plaque psoriasis showed positive outcomes, indicating improved symptoms and quality of life. Limitations include heterogeneous studies, sampling bias, and quality of evidence. This systematic review reveals limited and varied evidence for systemic antibiotic therapy efficacy in psoriasis treatment, while tonsillectomy emerges as a potentially beneficial antistreptococcal option, urging further well-designed, controlled studies with larger sample sizes and standardized protocols for better comparisons.

## Introduction

Psoriasis is a common skin condition affecting approximately 2% of the population [1]. The association between Streptococcus pyogenes infection and both guttate and plaque psoriasis has been well documented [2]. It is theorized that treating an associated streptococcal infection in a patient with psoriasis will lead to improvement of the psoriasis. However, the effectiveness of treating psoriasis patients who have concurrent streptococcal infection with systemic antibiotics or tonsillectomy is still a matter of debate [3]. Other reviews have been conducted to analyze anti-streptococcal therapy for psoriasis but have only included randomized controlled trials [4]. While randomized controlled trials are considered the gold standard for evaluating treatment efficacy, they have certain limitations, including strict inclusion and exclusion criteria that may limit the generalizability of the findings. Therefore, in this review, we aim to conduct a comprehensive analysis of studies of all designs which evaluate the effectiveness of antistreptococcal therapy in improving psoriasis.

## Methods and materials

### Literature search

This study was conducted in accordance with the Preferred Reporting Items for Systematic Reviews and Meta-Analyses (PRISMA) guidelines and registered in PROSPERO (338,776) [5]. PubMed, Scopus, and Embase databases were searched from their inception until August 14, 2022. The search strategy was validated by a qualified and experienced healthcare librarian. Two authors independently screened abstracts to determine eligibility for inclusion in the systematic review based on the criteria below. Any disagreements were resolved by a third author.

### Inclusion and exclusion criteria

All study designs were considered for inclusion. However, studies had to be original and include patients with any type of psoriatic lesions treated with any form of systemic antibiotics or tonsillectomy. Studies in languages other than English and studies without full text accessible were excluded.

### Data extraction

When applicable, the following data were collected from each published study: title, author, year of publication, country, study design, aim of study, randomization, number of participants, sex, mean age, participant withdrawals or exclusions, duration of participation, psoriasis type, psoriasis severity, intervention, dosage, duration of treatment, co-interventions, outcomes, evaluation, and comparison to control. Two authors independently extracted data.

### Risk of bias assessment

A risk of bias assessment was performed for each of the included articles. The Cochrane RoB 2.0 tool was used for RCTs, the ROBINS-I tool was used for non-randomized studies, the Newcastle–Ottawa Scale (NOS) for assessing the quality of nonrandomized studies in meta-analyses was used for case–control studies, the JBI Critical Checklist for Case Series was used for case series, and the JBI Critical Checklist for Case Reports was used for case reports [6–9]. Risk of bias assessment was conducted by one author and verified by a second author.

## Results

Our initial search of the literature yielded 2,630 non-duplicate studies for screening. After full-text screening, we narrowed our study pool to 50. Of these 50 studies, 38 studies evaluated the efficacy of systemic antibiotics, while 12 studies evaluated the efficacy of tonsillectomy, and two studies evaluated both (McMillin and Whyte). The studies consisted of 10 randomized controlled trials (RCTs) [10–19], 3 open-label studies [20–22], 1 crossover trial [23], 2 single-arm studies [24, 25], 1 prospective observational study [26], 1 case–control study [27], 1 retrospective questionnaire analysis [28], 2 cohort studies [29, 30], 13 case series [31–43], and 16 case reports [44–59].

### Efficacy of systemic antibiotic therapy

In total, 38 studies with a total of 1,369 patients investigated the efficacy of systemic antibiotic therapy in the treatment of psoriasis (Table 1).

**Table 1:** The efficacy of systemic antibiotics in the treatment of psoriasis

Authors	Study design	Patients *n*	Age (years) mean ± SD	Sex (F/M), *n*	Psoriasis type(s)	Psoriasis severity	Test for streptococcal infection	Patients w/o strep infection excluded?	Intervention(s)	Adjunct therapy	Outcome measure(s)	Result
Dogan et al. [10]	RCT	43	21	M: 43	Guttate psoriasis	–	All 43 patients included had confirmed streptococcal infection with ASO titer and/or β-hemolytic streptococcal culture	Yes	Group A (*n* = 15): no treatment Group B (*n* = 14): erythromycin 250 mg four times a day for 14 days Group C (*n* = 14): benzathine phenoxymethylpenicillin 50 000 IU/kg/day divided in three doses for 14 days	Emollients	PASI^a^	No statistically significant clinical improvement evaluated with PASI scores was detected in either treatment or control groups at the end of the treatment and during the follow-up periods
Dogra et al. [11]	RCT (Abstract)	100	–	–	Chronic plaque psoriasis	Moderate and severe	–	–	Group A (*n* = 50): penicillin 400 mg twice daily for 10 days, followed by 10 days off, repeat Group B (*n* = 50): placebo	–	PASI	Significant improvement in PASI score in Group A as compared to Group B Group A: basal mean PASI 32.42 ± 12.58 to 4.32 ± 5.16 at 6 months Group B: basal mean PASI 34.77 ± 11.48 to 32.72 ± 16.20 at 6 months
Grozdev et al. [12]	RCT (Abstract)	117	–	–	Guttate psoriasis, chronic plaque psoriasis	Moderate-to-severe	–	–	1: rifampin (guttate psoriasis, *n* = 82) 2: rifampin (chronic plaque psoriasis, *n* = 25) 3: placebo (guttate psoriasis, *n* = 10)	–	Reduction of PASI after 60 days	1: Rifampin was significantly more effective than placebo in guttate psoriasis group (*p* < 0.005). Improvement was statistically identical in patients with concomitant streptococcal infection and in those without concomitant streptococcal infection (p < 0.001) 2: In plaque psoriasis group, 12% of patients had PASI 75 on the 60th day of treatment
Grozdev et al. [13]	RCT (Abstract)	98	–	–	Guttate psoriasis with and without concomitant streptococcal infection (n = 76), chronic plaque psoriasis (n = 22)	Moderate-to-severe	–	–	1: rifampin 600 mg daily (*n* = 88) 2: placebo (*n* = 10)	Emollients	Achievement of PASI 75^b^	78% for guttate psoriasis with concomitant streptococcal infection 72% for patients without concomitant streptococcal infection 41% for moderate-to-severe plaque psoriasis 20% for placebo patients
Saxena et al. [14]	RCT	50	–	F: 18 M: 32	Chronic plaque psoriasis	Moderate-to-severe	ASO titer positive in 27 patients (16 from study group, 11 from control group) Throat culture results were equivocal	No	1: azithromycin 500 mg daily (*n* = 30) 2: vitamin C tablets (*n* = 20)	Mineral oil	Mean change ± SD of PASI	1: 21.65 ± 0.83 at 48th week 2: 0.40 ± 0.83 at 48th week *p* < .001
Tsankov et al. [15]	RCT	92	–	F: 48 M: 44	Guttate psoriasis	–	ASO titer Culture from the pharynx or vaginal smear	No	1: rifampin 600 mg daily (concomitant streptococcal infection, *n* = 39) 2: rifampin 600 mg daily (no evidence of streptococcal infection, *n* = 43) 3: placebo (*n* = 10)	Emollients	Mean reduction PASI	1: 75% 2: 71% 3: 39% The efficacy of rifampin compared with placebo was significantly higher (*p* < 0.005)
Tsankov et al. [16]	RCT	87	–	F: 42 M: 30	Eruptive psoriasis	–	ASO titer Pharynx and vaginal culture	No	1: rifampin 600 mg daily (concomitant streptococcal infection, *n* = 30) 2: rifampin 600 mg daily (no evidence of streptococcal infection, *n* = 42) 3: placebo (*n* = 15)	Emollients	PGA^c^	1: 50% remission, 23% marked improvement, 23% improvement, 3% no change 2: 43% remission, 31% marked improvement, 24% improvement, 2% no change 3: 13% remission, 20% marked improvement, 27% improvement, 40% no change
Vincent et al. [17]	RCT	20	–	F: 13 M: 7	Guttate psoriasis	–	Cultural or serologic evidence of β-hemolytic streptococcal colonization	Yes	A (*n* = 11): penicillin V or erythromycin 250 mg four times a day followed by placebo twice a day for the last 5 days of the 14 days B (*n* = 9): penicillin V or erythromycin 250 mg four times a day followed by rifampin 300 mg twice a day for the last 5 days of the 14 days	Emollients	PASI	No clinical change in psoriasis was detectable in either treatment group throughout the study
Caca-Biljanovska et al. [20]	Open-label	20	≥ 18	F: 11 M: 9	Guttate psoriasis	–	ASO titer positive in 4 patients from the treatment group (*n* = 10) and in 2 patients from the control group (*n* = 10) Nose and throat cultures were negative for all patients	No	1: penicillin + topical betamethasone dipropionate 0.05% cream and phototherapy (UVB) (*n* = 10) 2: topical betamethasone dipropionate 0.05% cream and phototherapy (UVB) (*n* = 10)	None	Change in mean PASI	1: From 5.66 ± 2.12 to 0.48 ± 0.79 after 6 weeks in the penicillin group 2: From 5.87 ± 2.47 to 0.96 ± 0.90 after 6 weeks in the group without penicillin No significant difference in the effectiveness of the two therapies (ANOVA; Fx = 1.402, df = 5, *p* > 0.05)
Polat et al. [21]	Open-label	60	1: 35.2 ± 12.8 2: 35.7 ± 14.7	F: 31 M: 29	Psoriasis vulgaris	–	ASO titer, results not stated Throat and urine culture, only 2 patients with positive culture in treatment arm (*n* = 36)	No	1: erythromycin and topical steroids (*n* = 36) 2: topical steroids (*n* = 24)	None	Mean PASI	Baseline PASI 11.58 ± 4.18 vs. 11.30 ± 4.93 (*p* = 0.815) End of treatment PASI 3.00 ± 2.13 vs. 4.87 ± 3.41 (*p* = 0.023) Mean difference in PASI 4.87 ± 3.41 vs. 6.43 ± 2.27 (*p* = 0.03)
Tsankov et al. [22]	Open-label	52	–	F: 30 M: 22	Guttate psoriasis	–	ASO titer Nasal and vaginal culture	No	1: rifampin 600 mg daily (evidence of concomitant streptococcal infection) 2: rifampin 600 mg daily (lack of concomitant streptococcal infection)	Emollients	Mean PASI	1: Decreased from 7.75 at baseline to 2.57 on day 60 2: Decreased from 9.13 at baseline to 2.96 on day 60 Improvement in the two groups was not significantly different (*p* < 0.001)
Ward et al. [23]	Crossover trial	60	52.4 ± 12.3	F: 48 M: 12	PPP	–	–	–	Clomocycline then placebo or placebo then clomocycline	Emulsifying ointment or dilute Betnovate 1:4 in petrolatum Six patients were left on topical steroids	Treatment preference	33% failed to complete treatment 37% did not respond to either treatment (no preference) 25% preferred clomocycline 3% preferred placebo 2% improved on both treatments (no preference)
Masood et al. [24]	Single-arm	200	–	–	Acute guttate psoriasis (*n* = 100), acute exacerbation of chronic psoriasis (*n* = 80), chronic plaque psoriasis (*n* = 20)	–	All patients tested with throat cultures and ASO titers, but results were not provided	–	Penicillin-V 250–500 mg every 6 h	Emollients	Clearance	Acute guttate psoriasis: 80% completely cleared, 6% partial improvement, 4% no improvement Acute exacerbations of psoriasis: 50% completely cleared, 17.5% partial improvement, 25% no improvement Chronic plaque psoriasis: 10% completely cleared, 20% partial improvement, 50% no improvement
Saxena et al. [25]	Single-arm	30	32.1 ± 13.0	F: 10 M: 20	Chronic plaque psoriasis	Moderate-to-severe	ASO titer positive in 50% (*n* = 15) Throat culture positive for group A streptococcus in 7% (*n* = 2)	No	IM benzathine penicillin biweekly for 6 months, and then monthly for total of 24 months	None	Mean PASI ± SD	From 32.76 ± 16.26 before initiation of therapy to 1.54 ± 4.86 after 48 weeks (*p* < 0.001)
Farrell et al. [27]	Case–control	230	–	F: 124 M: 106	Post-active phase streptococcal guttate psoriasis	–	ASO titer positive in all	Yes	1: single-course antibiotics^d^ (cases, *n* = 78) 2: control (*n* = 152)	All 78 cases and 152 (97.5%) controls received other (non-antibiotic) treatment 63 (81%) cases and 133 (85%) controls used topical corticosteroids with or without vitamin D analogues 50 (64%) cases and 98 (63%) controls underwent narrowband ultraviolet B therapy	Time to clearance	Mean time to clearance was 4.3 and 5.1 weeks in cases and controls, respectively In comparison to controls, antibiotic therapy did not significantly affect time to clearance (*χ*^2^ = 0.92, *p* = 0.82)
Bedi et al. [29]	Retrospective cohort	4	7.19 ± 3.16	–	Guttate psoriasis	–	ASO titer positive in all Throat culture positive in all	–	Penicillin 130 mg 3 times daily	Coal tar ointment	Resolution	All showed improvement within 1 week, but lesions often reappeared on stoppage of therapy after 2–4 weeks
Honig et al. [31]	Case series	4	6.8 ± 4.0	F: 1 M: 3	Guttate psoriasis	–	Perianal cultures all positive	Yes	Penicillin (*n* = 3) Erythromycin (*n* = 1)	–	Resolution	Psoriasis cleared in all patients after 14 to 35 days
Masood et al. [32]	Case series	60	–	–	Acute guttate psoriasis	–	ASO titer, results not stated Throat culture, results not stated	–	Rifampin 25 mg/kg body weight	Emollients	Clearance	43% completely cleared, 17% partially improved, 20% no clinical improvement, 20% did not report for follow up
McMillin et al. [33]	Case series	1 (Case 1)	11	M: 1	Guttate psoriasis	Severe	Recurrent streptococcal tonsillitis, testing not mentioned	–	Amoxicillin and amoxicillin/clavulanate	–	Resolution	Psoriasis did not resolve with medical therapy
Patrizi et al. [34]	Case series	5	7.4 ± 1.9	F: 2 M: 3	Guttate psoriasis	–	Streptococcal cultures all positive	–	Penicillin or erythromycin	–	Resolution	All patients had resolution of their guttate psoriasis
Rehder et al. [35]	Case series	1 (Case 4)	1	F: 1	Guttate psoriasis	–	Perirectal culture positive	–	Penicillin	–	Resolution	Psoriasis cleared in three weeks
Rosenberg et al. [36]	Case series	9	23.4 ± 14.5	F: 5 M: 4	Guttate psoriasis	–	All positive via throat/skin culture, ASO, anti-DNAase B, anti-hyaluronidase titers, or streptozyme titer	–	Rifampin + erythromycin or rifampin + penicillin	Emollients or “weak, previously used corticosteroids”	Response to treatment^e^	56% excellent, 44% good, and 0% less than good
Stokes et al. [37]	Case series	2	39.0 ± 13.0	M: 2	–	Severe	Stool and skin cultures	–	Terramycin	Milk injections, sevinon, sufathalidine, B12, niacin, UV lamp, anthralin, Desenex	Subjective resolution	One patient showed > 65% clearing on 6 weeks of Terramycin and one patient showed improvement within a month
Tsankov et al. [38]	Case series	10	–	F: 4 M: 6	Erythroderma (*n* = 7), pustular psoriasis (*n* = 2), and plaque psoriasis (*n* = 1)	Severe	ASO titer positive in 6 patients, throat culture positive in 6 patients	No	Rifampin 300 mg twice daily for 30–45 days	Emollients	Reduction of TBSA^f^	20% clinical remission, 20% great improvement, 20% moderate improvement, 40% without change
Whyte et al. [39]	Case series	1 (Case 4)	25	M: 1	Plaque psoriasis	–	ASO titer positive Throat culture positive	–	Penicillin + topical 2% coal tar ointment + daily ultraviolet light	Withdrawal from steroids, coal tar ointment, UV light	Resolution	Resolved by 90% in two weeks and no further psoriasis flares after one year
Banno et al. [44]	Case report	1	8	F: 1	Acute guttate psoriasis	–	ASO titer positive Pharyngeal culture positive	–	“Oral antimicrobials”	–	Resolution	Remission of disease and reduced ASO value
Belew-Noah et al. [45]	Case report	1	42	M: 1	Guttate psoriasis	–	ASO, anti-DNAase B, and anti-hyaluronidase titers all positive	–	Oral penicillin V, IM benzathine penicillin, and oral rifampin taken during the period of penicillin treatment	–	Resolution	Psoriasis completely cleared after four months of treatment
Cassandra et al. [46]	Case report	1	10	M: 1	Pustular psoriasis	Severe	ASO titer positive	–	Cephalexin and then penicillin V	Salicylic acid shampoo and clobetasol propionate lotion	Resolution	Full resolution one month after therapy
Garritsen et al. [47]	Case report	1	1	M: 1	Guttate psoriasis	–	Perianal culture positive	–	Phenethicillin 125 mg three times daily for 10 days	Mometasone ointment	Resolution	Psoriasis improved significantly after four weeks, and no relapse was observed after two months
Guidetti et al. [48]	Case report	1	57	F: 1	PPP	–	ASO titer positive	–	Amoxicillin 2 g per day for ten days	–	Resolution	Rapid cure of pustular lesions, and no relapses of the dermatosis at one-year follow-up
Herbst et al. [49]	Case report	1	4	M: 1	Guttate psoriasis	–	Perianal culture positive Streptozyme test positive	–	Penicillin	Topical fusidic acid for 2 weeks	Resolution	Improved dramatically
Hoffman et al. [50]	Case report	1	32	M: 1	Pustular psoriasis	–	ASO titer positive	–	Single intramuscular injection of benzathine penicillin	Topical amcinonide ointment	Resolution	Eruption resolved completely after one week
Horner et al. [51]	Case report	1	19	F: 1	Guttate psoriasis	–	Throat culture positive	–	Amoxicillin	Alclometasone cream, mometasone ointment, hydroxyzine, calcipotriene cream	Resolution	80% improvement after three weeks, flared again to initial severity, treated with another month of amoxicillin and had another recurrent flare, then tried calcipotriene cream twice a day for 2 weeks with satisfactory resolution
Maiolo et al. [52]	Case report	1	68	M: 1	Pustular psoriasis	–	Perianal culture positive	–	Clindamycin	Oral prednisolone, topical methylprednisolone aceponate, cephalexin, potassium permanganate soaks, clioquinol/betamethasone valerate, and acitretin	Resolution	Eruption resolved over several weeks
Pacifico et al. [53]	Case report	1	7	F: 1	Guttate psoriasis	–	ASO and anti-DNAase B titers positive Throat culture positive	–	Amoxicillin-clavulanate	None	Resolution	Skin cleared 30 days after starting therapy
Rasi et al. [54]	Case report	1	4	M: 1	Plaque psoriasis	–	Perianal culture positive	–	Amoxicillin	None	Resolution	Did not respond to therapy
Romano et al. [55]	Case report	1	12	M: 1	Guttate psoriasis	–	Perianal culture positive	–	Erythromycin	Mupirocin ointment	Resolution	Complete resolution after one month
Shelley et al. [56]	Case report	1	39	F: 1	Pustular psoriasis	–	Patient given streptococcal antigen	–	Clindamycin	–	Symptom control	Lesions well-controlled by oral clindamycin 150 mg four times daily

^a^0 = worse, 1 = no change; 2 = moderate improvement (partial reduction in scaling and/or erythema and/or infiltration), 3 = considerable improvement (significant reduction in all three variables), and 4 = clearing (almost no skin changes left, except residual erythema as in macular psoriasis). ^b^At least a 75% improvement in the Psoriasis Area and Severity Index. ^c^Remission = no erythemous and squamous plaques; residual discoloration may be present, Marked improvement = single erythemous plaques are present; no desquamation; no infiltration; hypo- or hyperpigmented post-lesional macules are present, Improvement = more than 50% of the plaques are in remission; erythemous plaques with infiltration and desquamation; hypo- or hyperpigmented post-lesional macules are present, No change = status idem, Worse = appearance of new erythemous and squamous plaques; dissemination and generalization of the lesions. ^d^31 (40%) patients were prescribed a penicillin antibiotic, 22 (28%) macrolide, 20 (25.5%) cephalosporin and 5 (6.5%) other antibiotic (2 tetracycline, 2 fluoroquinolone and 1 lincosamide). ^e^Excellent response = 95% to 100% disappearance of lesions, good response = 80% to 95% improvement; less than good response < 80% improvement. F TBSA = total body surface area. This was estimated by the rule of nines, diminution of scaling, erythema, and thickness of erythroderma and plaque-type psoriasis, and disappearance of pustules in pustular psoriasis

ASO = antistreptolysin-O

### Penicillins/aminopenicillins

Twenty-one studies assessed penicillins/aminopenicillins' effectiveness. Two RCTs [10, 11] investigated penicillin's role in psoriasis treatment. Dogan et al. [10] found no significant PASI score differences in guttate psoriasis patients treated with benzathine phenoxymethylpenicillin (n = 14), erythromycin (n = 14), or no treatment (n = 15). All 43 patients had confirmed streptococcal infection. Conversely, Dogra et al. [11] demonstrated notable PASI improvement in moderate-to-severe chronic plaque psoriasis (n = 50) with penicillin treatment (400 mg twice daily for 12 weeks) compared to placebo (n = 50), without testing for streptococcal infection. An open-label study [20] by Caca-Biljanovska et al. showed no significant difference in mean PASI change for guttate psoriasis patients treated with penicillin (n = 10) alongside steroids and phototherapy, compared to without penicillin (n = 10). Two single-arm trials [24, 25] found penicillin effective: Masood et al. [24] in acute guttate psoriasis (n = 100; 80% cleared) and exacerbations (n = 80; 50% cleared), and Saxena et al. [25] in chronic plaque psoriasis (n = 30), with positive ASO titers in 50% and streptococcus cultures in 7%. Bedi et al. [29] found improvement in guttate psoriasis (n = 4) with penicillin (130 mg thrice daily) and confirmed throat streptococcal infection, but lesions often recurred post-treatment. Five [31, 33, 35, 36, 39] of six case series showed positive penicillin outcomes, while one [34] reported ineffectiveness for guttate psoriasis. Eight case reports [45–51, 53] noted psoriasis resolution with penicillin, amoxicillin, or amoxicillin-clavulanate, but one [54] didn't respond to amoxicillin. McMillin et al.’s [33] case series lacked streptococcal testing. Remaining case series and reports confirmed streptococcal infection by culture or serology.

### Rifampin

Ten studies assessed rifampin's efficacy for improving psoriasis. Grozdev et al. [12] found rifampin significantly more effective than placebo in treating guttate psoriasis (p < 0.005), regardless of streptococcal infection. Notably, 12% of chronic plaque psoriasis patients achieved PASI 75 after 60 days. In another study by Grozdev et al. [13], 78% of guttate psoriasis patients with streptococcal infection achieved PASI 75 with rifampin vs. 72% without, compared to 41% with chronic plaque psoriasis and 20% with placebo. Tsankov et al. [15] demonstrated rifampin's superiority over placebo in reducing mean PASI for guttate psoriasis (p < 0.005), irrespective of streptococcal infection. Tsankov et al. [16] had similar findings using Physician Global Assessment (PGA). Vincent et al. [17] showed no clinical change with rifampin or placebo combined with penicillin V/erythromycin for guttate psoriasis (n = 20), despite streptococcal colonization evidence. In an open-label trial, Tsankov et al. [22] showed PASI improvement with rifampin, regardless of streptococcal infection (p < 0.001). Case series [32, 36, 38], and a case report [45] displayed positive responses to rifampin in streptococcal-infected psoriasis patients. Masood et al. [32] included 60 patients without streptococcal testing. Tsankov et al. [38] included 10 patients, 6 with confirmed streptococcal infection. The remaining cases had confirmed streptococcal infection by culture/serology.

### Other systemic antibiotics

Two randomized controlled trials (RCTs) assessed macrolides' efficacy in improving psoriasis. In an RCT by Dogan et al. [10], erythromycin showed no significant improvement in PASI. Another RCT by Saxena et al. [14] examined azithromycin (n = 30) and vitamin C tablets (n = 20) in chronic plaque psoriasis patients. After 48 weeks, the azithromycin group displayed a notable mean PASI change (21.65 ± 0.83) versus the vitamin C group (0.40 ± 0.83) (p < 0.001). ASO titers were > 200 IU/ml in 54% of patients. Polat et al. [21] conducted an open-label trial comparing erythromycin and topical steroids to steroids alone. The treatment group (n = 36) showed a statistically significant mean PASI change (8.57 ± 2.90) compared to the control group (n = 24) (p = 0.03). Streptococcal culture was positive for 2 patients in the treatment arm. In a crossover trial by Ward et al. [23] on palmoplantar pustulosis patients (n = 60), clomocycline and placebo were examined, with 37% non-response, 25% preference for clomocycline, and 3% for placebo. Streptococcal infection was not reported. Farrell et al. [27] conducted a case–control study on guttate psoriasis patients (n = 230) treated with antibiotics (n = 78) versus controls (n = 152) with ASO titers > 200 IU/ml in all. Various antibiotics were used, including penicillin (40%), macrolide (28%), cephalosporin (25.5%), and others (6.5%). Antibiotics did not significantly affect time to clearance compared to controls (χ2 = 0.92, p = 0.82). Four case series [31, 34, 36, 37] on erythromycin/terramycin for streptococcal-induced guttate psoriasis reported positive outcomes. Additionally, five case reports [44, 46, 52, 55, 56] demonstrated positive results for cephalexin, clindamycin, and erythromycin. All cases confirmed streptococcal infection by culture or serology.

### Efficacy of tonsillectomy

In 14 studies involving 409 patients (Table 2), the efficacy of tonsillectomy for psoriasis treatment was assessed. Thorleifsdottir et al. [18] conducted an RCT comparing tonsillectomy (n = 15) with control (n = 14) in chronic plaque psoriasis patients with a history of psoriasis exacerbation after throat infections. Without streptococcal testing, 13 of 15 participants showed 30–90% reduction in PASI score, with 50% lesion reduction in 9 of 15. The control group demonstrated no improvement; 86% used topical treatment vs. 27% in the treatment group. Similarly, Thorleifsdottir et al. [19] noted reduced Psoriasis Disability Index (PDI) in treatment over time (p = 0.026) and vs. controls (p = 0.037). Ueda et al. [26] prospectively studied post-tonsillectomy pustulosis palmaris et plantaris (PPP, n = 33) using skin severity scores (SSS); post-tonsillectomy mean SSS was 3.5 ± 2.2. Nyfors et al. [28] retrospectively analyzed 74 psoriasis vulgaris patients; after tonsillectomy, 32% cleared, 39% improved, 22% unchanged, 7% worsened. Takahara et al. [30] assessed 138 PPP patients post-tonsillectomy for SSS (n = 138) and Palmoplantar Pustulosis Psoriasis Area Severity Index (PPPASI)(n = 80); 44% and 78% showed complete improvement at 12 and 24 months, respectively, with 70% and 95% seeing 80% + improvement. Fifty percent of patients had positive ASO antibodies. Six case series [33, 39–43] demonstrated tonsillectomy's efficacy, mainly in streptococcal-associated guttate and chronic plaque psoriasis. Two case reports [57, 58] detailed guttate psoriasis patients; psoriasis resolved post-tonsillectomy. One case report [59] investigated plaque psoriasis; PASI reduced from 26.8 to 1 after tonsillectomy. The remaining case series and reports included patients with confirmed streptococcal infection via culture or serology.

**Table 2:** The efficacy of tonsillectomy in the treatment of psoriasis

Authors	Study design	Patients *n*	Age (years) mean ± SD	Sex (F/M), *n*	Psoriasis type(s)	Psoriasis severity	Test for streptococcal infection	Patients w/o strep infection excluded?	Intervention(s)	Adjunct therapy	Outcome measure(s)	Result
Thorleifsdottir et al. [18]	RCT	29	35.3 ± 9.9	F: 20 M: 9	Chronic plaque psoriasis	–	History of psoriasis exacerbation during or shortly after throat infections, but no streptococcal testing	–	1: Tonsillectomy (*n* = 15) 2: Control (*n* = 14)	None	PASI	1: 13 of 15 (87%) participants showed improvement ranging from 30–90% reduction of PASI score, and 9 of 15 (60%) reached 50% reduction in skin lesions during the study 2: No corresponding clinical improvement observed
Thorleifsdottir et al. [19]	RCT	29	35.3 ± 9.9	F: 20 M: 9	Plaque psoriasis and history of streptococcal-associated psoriasis exacerbations	Moderate-to-severe	History of sore throat-associated psoriasis exacerbation, but no streptococcal testing	–	1: Tonsillectomy (*n* = 15) 2: Control (*n* = 14)	Moisturizers	HRQoL^a^, assessed by PDI^b^ and PLSI^c^	1: Improvement in HRQoL with a significant decrease in the mean PDI, both with time (*p* = 0.026) and compared with the controls (*p* = 0.037). Significantly decreased PLSI, both with time (*p* < 0.001) and compared with the controls (*p* = 0.002) 2: No corresponding changes in PDI or PLSI
Ueda et al. [26]	Prospective observational study	33	–	F: 22 M: 11	PPP^d^	–	ASO titer, positive in 30% (*n* = 10) of patients α-Streptococci were identified in all patients in both groups by microbiological examination	Yes	All patients received tonsillectomy due to recurrent episodes (> 3 × in 1 year) of acute tonsillitis 1: PPP group 2: Non-PPP without skin disease	–	Changes in skin severity score^e^ after tonsillectomy	1: Mean skin severity score 3.5 ± 2.2 2: N/A
Nyfors et al. [28]	Retrospective questionnaire analysis	74	14.2	F: 56 M: 18	Psoriasis vulgaris	–	Recurrent tonsillitis, but no streptococcal testing	–	Tonsillectomy	Emollients	Clearance	32% cleared, 39% improved, 22% unchanged, 7% worsened
Takahara et al. [30]	Retrospective cohort	138	–	F: 105 M: 33	PPP	–	ASO titer, 50% of patients were positive	No	Tonsillectomy	–	Skin severity score and PPPASI^f^	Skin severity score (n = 138): At 12 and 24 months after tonsillectomy, the PPP lesion disappeared in 38% and 66% of patients, respectively, and improved 80% or more in 71% and 95% of patients, respectively PPPASI (n = 80): At 12 and 24 months after tonsillectomy, 100% improvement in PPPASI was seen in 44% and 78% of patients, respectively, and 80% or more improvement in PPPASI was seen in 70% and 95% of patients, respectively
Hone et al. [40]	Case series	13	17.0	F: 12 M: 1	Guttate psoriasis (*n* = 6), chronic plaque psoriasis (*n* = 7)	–	Recurrent tonsillitis, but no streptococcal testing	–	Tonsillectomy	–	Clearance	Total clearance in 53% of patients, significant improvement in 23%, and unchanged in 23%
McMillin et al. [33]	Case series	2	8.0	F: 1 M: 1	Guttate psoriasis	Severe	No streptococcal testing, but referenced recurrent streptococcal pharyngitis	–	Adenotonsillectomy	–	Clearance	Both patients experienced a significant improvement in their psoriasis, and both were completely free of psoriatic outbreaks after 16 months
Saita et al. [41]	Case series	2	9.0	F: 2	Guttate psoriasis	Severe	Tonsillar culture positive Antistreptokinase titer positive ASO titer positive	–	Tonsillectomy	Oral antibiotics and topical corticosteroids	Clearance	Both cases healed almost completely within two months after surgery
Thorleifsdottir et al. [42]	Case series	56	–	–	Guttate psoriasis and plaque psoriasis	–	Streptococcal infection confirmed by throat culture or rapid antigen detection test	No	Tonsillectomy	–	Improvement	48% reported improvement after tonsillectomy, while 52% were unsure or reported no improvement
Thorleifsdottir et al. [43]	Case series	28	33.6 ± 9.8	F: 22 M: 6	Plaque psoriasis	Moderate-to-severe	Diagnosed by a physician, throat culture, or rapid strep test; positive in 61% (*n* = 17) of patients	Yes	Tonsillectomy	Topical corticosteroids, vitamin-D analog creams, phototherapy, or methotrexate before and/or after tonsillectomy	PASI	Mean reduction in PASI scores of 4.4 points at 24 months
Whyte et al. [39]	Case series	2 (Cases 2 & 3)	22.0	F: 1 M: 1	Guttate psoriasis	–	ASO titer positive Throat culture positive in one	–	Tonsillectomy	–	Resolution	One patient had no further guttate flares one year after surgery and one patient had very mild plaque psoriasis on the elbows and knees one year after surgery
Cohn et al. [57]	Case report	1	26	M: 1	Guttate psoriasis	–	History of “streptococcal tonsillitis”, but no streptococcal testing	–	Tonsillectomy	Levofloxacin and topical steroids before tonsillectomy	Resolution	The patient's guttate psoriasis completely resolved after tonsillectomy
Loyal et al. [58]	Case report	1	19	F: 1	Guttate psoriasis	–	Recurrent tonsillitis and “streptococcal pharyngitis”, but no streptococcal testing	–	Adenotonsillectomy	Topical corticosteroids, apremilast, UVB, salicylic acid shampoo before tonsillectomy	Resolution	The psoriasis had cleared completely three months after surgery
Simoes et al. [59]	Case report	1	39	M: 1	Plaque psoriasis	Severe	Streptococcus rapid immunoassay positive	–	Tonsillectomy	Phototherapy, topical steroids and calcipotriol, and acitretin before tonsillectomy	PASI	From PASI 26.8 pre-operatively to PASI 1 after 4 months

^a^HRQoL = health-related quality of life; ^b^PDI = Psoriasis Disability Index; ^c^PLSI = Psoriasis Life Stress Inventory; ^d^PPP = pustulosis palmaris et plantaris; ^e^The skin severity score prior to tonsillectomy in each patient was defined as 10 points, regardless of the skin condition. The skin severity score at 3 months after tonsillectomy was rescored on hospital chart by a dermatologist and/or an otolaryngologist. 0 = completely effective, 1 or 2 = markedly effective, 3–5 = effective, 6–8 = partially effective, 9–11 = not effective, 12 +  = worsened; ^f^Palmoplantar Pustulosis Area and Severity Index

## Discussion

The findings of this review suggest a potential role of systemic antibiotic therapy in the treatment of psoriasis, particularly guttate psoriasis, with or without confirmed streptococcal infection. Among 38 studies assessing systemic antibiotics' efficacy in psoriasis treatment, penicillins/aminopenicillins were most studied (21 studies). In the largest RCT, Dogra et al. [11] demonstrated significant improvement in PASI scores with penicillin treatment compared to placebo. Two studies [24, 25] without control arms reported marked improvement in guttate psoriasis with penicillins. The effect size of the improvement in outcomes after treatment with penicillins was modest in the largest RCT, which may explain why the two smaller studies [10, 20] with controls were not powered to detect a difference in outcomes. Additional larger studies with control groups are needed to confirm penicillin antibiotics' benefit in psoriasis treatment. Studies should stratify outcomes based on confirmed streptococcal infection presence, providing insight into antibacterial or anti-inflammatory effects.

Rifampin, evaluated in ten studies (including five RCTs), demonstrated effectiveness in treating psoriasis, particularly in guttate psoriasis. The RCTs conducted by Grozdev et al. [12, 13] both reported positive outcomes, with rifampin showing superiority over placebo in improving PASI scores. However, both studies showed no statistical difference in improvement when patients with confirmed streptococcal infection were compared to those without. Likewise, the RCTs by Tsankov et al. [15, 16] showed significant improvement in PASI and PGA respectively for psoriasis patients treated with rifampin compared to placebo. In both studies, there was no significant difference in the response to rifampin comparing patients with guttate psoriasis with and without concomitant infection. This suggests that rifampin's positive outcomes are independent of a psoriasis patient's concurrent streptococcal infection. One possible rationale behind this independent influence could be attributed to rifampin's anti-inflammatory attributes rather than its antimicrobial properties [60]. In dermatology, antibiotics, especially tetracyclines, are extensively employed for their anti-inflammatory rather than antibacterial traits [61]. Although primarily used to treat conditions like acne and hidradenitis suppurativa, these anti-inflammatory effects could potentially yield benefits in psoriasis as well.

Macrolides, evaluated in two RCTs and one case–control study, showed mixed results. Azithromycin demonstrated significant PASI improvement [14], while erythromycin's effect was inconclusive due to small sample sizes [10] and potential confounding by concurrent phototherapy [27].

Tonsillectomy's potential as a treatment for psoriasis was assessed across 14 studies, indicating favorable effects on patients with chronic plaque psoriasis and guttate psoriasis. Notably, Thorleifsdottir et al. [18] conducted an RCT revealing improved PASI scores in chronic plaque psoriasis patients post tonsillectomy. Additionally, another study by Thorleifsdottir et al. [19] exhibited a noteworthy reduction in Psoriasis Disability Index (PDI), indicating improved quality of life. While streptococcal infection confirmation was lacking, patients in these studies showed psoriasis exacerbation post throat infections. Observational studies demonstrated psoriasis symptom amelioration after tonsillectomy in cases of psoriasis vulgaris and recurrent tonsillitis. Similarly, Ueda et al. [26] found reduced mean SSS in PPP patients, and Takahara et al. [30] noted significant SSS and PPPASI score improvements at 12- and 24-months post tonsillectomy. Positive outcomes were also reported in case series and reports.

The evidence compiled in this review is mixed. Several studies demonstrated improved outcomes with various systemic antibiotics, especially in patients with guttate psoriasis. However, these studies exhibited significant variability in the antibiotics and dosages used. Future studies should compare the efficacy of varying doses of different antibiotics to identify an optimal treatment regimen for patients with guttate psoriasis. Furthermore, these studies should compare outcomes in patients with and without concurrent streptococcal infection. Although limited to studies with rifampin, current studies show no difference in benefit from antibiotics between patients with and without concurrent confirmed streptococcal infection. It may be the case that patients with guttate psoriasis benefit from antibiotic treatment regardless of their streptococcal infection status, making the common practice of testing for streptococcal infection in these patients unnecessary. When considering the repeated use of antibiotics for patients with recurrent streptococcal infections, it is important to carefully evaluate and weigh the risk of developing antibiotic resistance [62]. The studies discussed in this review that assess the efficacy of tonsillectomy in patients with psoriasis suggest that it is beneficial in improving outcomes. Further studies are warranted to determine at what threshold a patient with psoriasis and recurrent streptococcal infections should be considered for tonsillectomy. Given the morbidity associated with tonsillectomy, it is important to weigh the risks of a tonsillectomy against the potential benefit to be gained in improving a patient’s psoriasis.

Several limitations warrant consideration when interpreting the findings from this systematic review. Firstly, the included studies displayed heterogeneity in study design, patient attributes, treatment protocols, and outcome gauges. While enhancing generalizability, this heterogeneity limits the strength of assertions regarding the effectiveness of individual antistreptococcal treatments within distinct patient populations. Secondly, evident sampling bias arises from tonsillectomy studies solely encompassing patients with recurrent streptococcal infections, whereas certain antibiotic studies scrutinized patients lacking confirmed streptococcal infections. Thirdly, the quality of evidence exhibited variation among the encompassed studies, primarily consisting of observational studies and case reports. This limitation highlights the need for larger, controlled studies of antistreptococcal treatment in psoriasis.

## Conclusion

This systematic review compiles the evidence for efficacy of antistreptococcal treatments, specifically systemic antibiotics and tonsillectomy, in the management of psoriasis. The findings suggest that systemic antibiotic therapy improves outcomes in psoriasis, especially guttate psoriasis and is not dependent on the presence of streptococcal infection. The antibiotic regimens used varied significantly, including penicillins, rifampin, and macrolides. Further research is required to identify an optimal systemic antibiotic treatment regimen for patients with psoriasis. Tonsillectomy showed efficacy in improving psoriasis and quality of life in patients in multiple studies. Further studies should delineate the patient population whose benefit from tonsillectomy outweighs the procedures associated risks.
